# Biological Detoxification of Aflatoxin B1: A Systematic Review of Microbial and Enzymatic Strategies, Mechanisms, and Applications in Food and Feed Systems

**DOI:** 10.3390/toxins18070313

**Published:** 2026-07-18

**Authors:** Sarra Rafai, Lara Manyes, Ana Moreno, Alessandra Cimbalo, Giuseppe Meca, Victor Dopazo

**Affiliations:** Laboratory of Food Chemistry and Toxicology, Faculty of Pharmacy, University of Valencia, Av. Vicent Andrés Estellés S/n, 46100 Burjassot, Spain; sarra.rafai@uv.es (S.R.); lara.manyes@uv.es (L.M.); ana.moreno@uv.es (A.M.); alessandra.cimbalo@uv.es (A.C.); giuseppe.meca@uv.es (G.M.)

**Keywords:** aflatoxin B1, biological detoxification, biodegradation, biotransformation, lactic acid bacteria, yeasts, laccase, microbial detoxification, food safety

## Abstract

Contamination of food and feed by aflatoxin B1 (AFB1) remains a major global concern due to its toxicity, carcinogenicity, and persistence in the food chain. Environmental and climatic pressures continue to favor aflatoxigenic fungal contamination, highlighting the need for effective and sustainable detoxification strategies. Biological detoxification has emerged as a promising alternative to conventional physical and chemical treatments. This systematic review, conducted following PRISMA guidelines, summarizes microbial and enzymatic approaches for AFB1 detoxification, focusing on bacteria, yeasts, and microbial enzymes. The literature shows a predominance of bacterial systems, especially lactic acid bacteria and Bacillus species, mainly acting through adsorption, fungal growth inhibition, and suppression of aflatoxin biosynthesis. Yeasts, although less represented, also showed promising detoxification capacities through adsorption and biodegradation-related mechanisms. Enzymatic systems achieved the highest efficiencies, particularly oxidative enzymes such as laccases and dye-decolorizing peroxidases, often exceeding 90% detoxification under optimized conditions. However, industrial application remains limited by laboratory-scale validation, variability among protocols, incomplete toxicological assessment of degradation products, and limited evidence in complex food and feed matrices.

## 1. Introduction

AFB1 is widely recognized as one of the most hazardous mycotoxins contaminating food and feed worldwide. It is produced predominantly by toxigenic *Aspergillus* species, particularly *Aspergillus flavus* and *Aspergillus parasiticus*, and is frequently detected in cereals, nuts, oilseeds, spices, and other staple commodities intended for human and animal consumption. Contamination can occur both in the field and during post-harvest handling or storage, especially under warm and humid environmental conditions that favor fungal proliferation and toxin biosynthesis. As a result, AFB1 remains a persistent challenge throughout the food chain and continues to compromise food safety, public health, and international trade [1].

The concern surrounding AFB1 has intensified further in recent years because environmental and climatic pressures increasingly favor aflatoxigenic fungi. Drought stress, elevated temperatures, fluctuations in humidity, insect infestation, plant stress, and inadequate storage infrastructure all contribute to fungal colonization and enhanced aflatoxin accumulation [2,3]. Current evidence also indicates that climate change may extend the geographic distribution of aflatoxin contamination into regions previously considered at lower risk, thereby increasing the urgency of both preventive and post-contamination control strategies [4].

Among the known aflatoxins, AFB1 is considered the most toxic and the most relevant from a carcinogenic perspective. Its toxicological significance is largely linked to hepatic bioactivation by cytochrome P450 enzymes, which convert AFB1 into the highly reactive AFB1-8,9-epoxide metabolite [5]. This reactive intermediate forms adducts with DNA and proteins, promotes oxidative stress, disrupts cellular homeostasis, and initiates mutagenic events strongly associated with hepatocellular carcinoma. In accordance with this evidence, aflatoxins have been classified by the International Agency for Research on Cancer (IARC) as carcinogenic to humans (Group 1) [6]. Beyond carcinogenicity, chronic exposure to AFB1 has also been associated with hepatotoxicity, immunotoxicity, impaired growth, and broader metabolic disturbances, particularly in vulnerable populations exposed to low but repeated doses [7].

Despite the establishment of regulatory limits and monitoring frameworks by major authorities, including the European Commission and the United States Food and Drug Administration, AFB1 contamination remains difficult to control in practice. Regulatory action levels and maximum limits are indispensable tools for risk management, but they do not eliminate the environmental, agricultural, and storage-related factors that drive contamination. Consequently, AFB1 concentrations continue to exceed permissible levels in many settings, particularly in regions where climatic stress, insufficient drying practices, and inadequate storage conditions facilitate fungal persistence and toxin production [8,9].

To mitigate AFB1 contamination, numerous interventions have been explored and are generally classified as physical, chemical, and biological approaches. Physical strategies include sorting, washing, peeling, thermal treatment, irradiation, and the use of adsorbent materials, whereas chemical methods involve compounds capable of modifying or degrading aflatoxin structures [10]. However, these approaches often present significant limitations, including incomplete detoxification, potential formation of undesirable by-products, loss of nutritional or sensory quality, matrix-dependent efficacy, and economic or operational constraints [11]. These drawbacks have stimulated increasing interest in safer, more sustainable, and food-compatible detoxification strategies.

Within this context, biological detoxification has emerged as one of the most promising alternatives for AFB1 mitigation. Historically, interest in microbial aflatoxin detoxification dates back to 1966, when Ciegler et al. first reported that microorganisms were capable of detoxifying aflatoxin and identified *Flavobacterium aurantiacum* as an active strain [12]. Subsequent studies describing microbial conversion products of AFB1 further demonstrated that biological detoxification may involve not only physical removal but also true chemical transformation of the toxin molecule [13]. These pioneering findings established the conceptual basis for modern research on microbial and enzymatic AFB1 detoxification.

Current biological detoxification strategies rely on microorganisms such as bacteria, yeasts, and filamentous fungi, as well as isolated enzymes, to reduce AFB1 through adsorption, inhibition of fungal growth and toxin biosynthesis, biotransformation, or direct enzymatic degradation. Compared with conventional chemical or physical interventions, biological approaches are generally regarded as milder and more compatible with food and feed systems because they may better preserve nutritional value and organoleptic properties while reducing environmental burden [14]. Recent literature particularly highlights the growing relevance of probiotic bacteria, food-grade yeasts, recombinant enzymes, and immobilized biocatalysts as innovative tools for AFB1 control [15].

At the same time, the field remains under active development, and several important knowledge gaps persist. Reported detoxification efficacy varies substantially depending on the microorganism or enzyme selected, reaction conditions, exposure time, toxin concentration, food or feed matrix, and analytical method used to verify AFB1 reduction. In addition, although many studies report the formation of AFB1 transformation products, their reduced toxicity is often not directly demonstrated, and concerns remain regarding the complete toxicological characterization of these metabolites, the reproducibility of results in complex real matrices, and the feasibility of large-scale industrial implementation [16]. Recent reviews therefore call for stronger mechanistic validation, more rigorous safety assessment, and improved translation from laboratory-scale findings to practical food and feed applications [17].

Within this framework, the present systematic review aims to provide a comprehensive synthesis of current knowledge on the biological detoxification of AFB1. Particular emphasis is placed on the mechanisms involved, the principal microbial and enzymatic agents investigated, their reported detoxification efficacy in food and feed systems, and the practical limitations that still restrict broader application. By integrating recent findings, this review seeks to clarify the potential of biological detoxification as a sustainable and scientifically robust strategy to reduce AFB1 exposure and improve food and feed safety.

## 2. Results

### 2.1. Bacterial Systems Involved in AFB1 Detoxification by Adsorption, Binding, or Inhibition of Toxin Biosynthesis

A wide diversity of bacterial strains has been investigated for AFB1 detoxification through adsorption, cell-surface binding, antagonism toward aflatoxigenic fungi, or suppression of aflatoxin biosynthesis. The collected studies mainly involved lactic acid bacteria, *Bacillus* spp., *Pseudomonas*, *Rhodococcus*, and *Streptomyces*, highlighting the predominance of food-associated and environmentally derived bacteria in non-enzymatic mitigation strategies (Table 1a). Depending on the strain and experimental model, the reported efficiencies ranged from modest adsorption capacities to complete inhibition of AFB1 production.

Among the most recurrent genera, *Bacillus* species showed particularly strong anti-aflatoxigenic performance. *Bacillus amyloliquefaciens* WF2020 completely inhibited AFB1 production during co-culture with *Aspergillus flavus* under liquid culture conditions [18]. Similarly, *B. amyloliquefaciens* YUAD7 significantly inhibited fungal growth and totally suppressed AFB1 production in PDB co-culture assays [19]. In an additional model, the same strain achieved 91.7% detoxification in liquid medium and more than 85% reduction in food matrices, indicating multifunctional activity combining binding and extracellular degradation mechanisms [20]. *Bacillus subtilis* E11 also displayed strong antagonistic potential, inhibiting *A. flavus* growth by approximately 64% and reducing AFB1 by up to 81.34% [22].

Adsorption-based detoxification was highly represented among lactic acid bacteria. *Lacticaseibacillus rhamnosus* GG reached an adsorption value of 97.74% under optimized conditions using heat-treated cells, while desorption remained ≤3% after simulated digestion [35]. *Limosilactobacillus fermentum* also showed high affinity for AFB1, with adsorption values of 87.30% for live cells and 70.49% for heat-treated cells [37]. In contrast, rapid short-contact assays using *Lactiplantibacillus pentosus* TV3 and *Pediococcus acidilactici* OR83 yielded lower adsorption values of 11.5% and 7.6%, respectively [26,38].

Several studies confirmed that adsorption efficiency was strongly strain-dependent. Badji et al. reported AFB1 adsorption ranging from 25% to 80% for *Enterococcus faecium*, *Enterococcus durans*, and *Lactobacillus plantarum*, with the highest activity observed in nonviable cells [25]. Similarly, Asurmendi et al. found values between 37.6% and 70.7% for bacterial isolates recovered from brewer’s grains [40], while Lemmetty et al. described reductions between 16% and 71% for food-derived lactic acid bacteria depending on pH and strain identity [36].

Some systems combined adsorption with marked antifungal or anti-aflatoxigenic activity. Møller et al. reported that selected lactic acid bacteria completely inhibited AFB1 production in *Aspergillus parasiticus* while simultaneously adsorbing 40–70% of the toxin [28]. Simões et al. further showed that strains isolated from naturally fermented Brazilian table olives completely inhibited *A. flavus* growth, achieved 100% AFB1 detoxification at the highest concentration of cell-free supernatant, and adsorbed 48–51% of residual toxin [29]. Yun et al. also demonstrated that *Pediococcus*, *Weissella*, and *Lactobacillus* strains from Korean Nuruk significantly reduced AFB1 production through combined inhibition of biosynthesis, adsorption, and production of antifungal metabolites [39].

Other non-lactic acid bacteria also demonstrated promising binding properties. Bacterial isolates from the rice weevil gut reduced AFB1 by 48.9–84.2%, depending on the strain tested [23]. *Bacillus* sp. MA82 increased adsorption from 45% at time zero to 75% after 24 h, indicating progressive toxin binding over time [21]. *Rhodococcus turbidus* PD630 also reached 93.04% detoxification after 72 h through adsorption-dominated interactions [42].

Actinomycete-based inhibition models were effective. *Streptomyces exfoliatus* completely inhibited AFB1 production when applied as culture filtrate at ≥20% (*v*/*v*) [43]. Selected *Streptomyces* isolates reduced residual AFB1 to 6% while strongly suppressing fungal growth [44].

Collectively, the studies summarized in Table 1a demonstrate that bacterial detoxification systems are highly versatile and rely mainly on cell-wall adsorption, fungal growth suppression, and inhibition of aflatoxin biosynthesis. Among the tested microorganisms, *Bacillus* spp. and lactic acid bacteria were the most frequently investigated and repeatedly showed substantial AFB1 reduction under their respective experimental conditions.

### 2.2. Bacterial and Selected Fungal Systems Involved in AFB1 Biodegradation and Biotransformation

Direct biodegradation and biotransformation of AFB1 have increasingly been investigated using predominantly bacterial strains, together with selected fungal isolates, capable of converting the toxin into structurally modified products or markedly reducing its concentration. In contrast to adsorption-based approaches, these strategies mainly relied on extracellular enzymes, secreted metabolites, proteinaceous fractions, or active metabolic conversion pathways. The reported studies involved microorganisms isolated from fermented foods, soil, animal-derived sources, silages, marine environments, and food-associated ecosystems, highlighting the ecological diversity of AFB1-detoxifying microorganisms (Table 1b).

Among the most effective bacterial systems, several *Bacillus* strains demonstrated remarkable detoxification performance. *Bacillus amyloliquefaciens* WF2020 reduced AFB1 by more than 80% after 72 h under liquid culture conditions, with degradation associated with extracellular enzymatic activity [18]. *Bacillus amyloliquefaciens* YUAD7 decreased AFB1 in artificially contaminated alfalfa silage by 99.7%, with a final residual concentration of only 1.7 µg/kg after 48 days [19]. *Bacillus albus* YUN5 also exhibited strong activity, reaching 76.28% detoxification through non-proteinaceous metabolites present in the cell-free supernatant [48], whereas *Bacillus licheniformis* QT338 achieved 61.03% reduction after 72 h [49].

Other bacterial genera similarly showed substantial biodegradation capacities. *Burkholderia contaminans* BC11-1 reduced AFB1 by 90% despite physical separation from the toxin through a membrane barrier, indicating detoxification mediated exclusively by diffusible extracellular metabolites [50]. *Burkholderia* sp. XHY-12 achieved 85.2% detoxification through extracellular enzymatic activity during liquid fermentation [51]. Similarly, *Enterococcus faecium* HB2-2 reduced AFB1 by 90.0% under optimized alkaline conditions, with the fermentation supernatant showing the highest activity and LC–MS confirming the formation of degradation products [52].

Several *Pseudomonas* strains were also highly efficient under the evaluated conditions. *Pseudomonas fluorescens* SZ1 reached up to 99% AFB1 detoxification after 72 h, with activity attributed to extracellular proteinaceous components [59]. Likewise, *Pseudomonas knackmussii* AD02 reduced AFB1 by 88.85–90.0% over a concentration range of 20–500 ng/mL, confirming robust degradation efficiency across variable toxin loads [60].

Biotransformation-based systems additionally provided evidence of structural modification. *Kocuria rosea* strain 13, isolated from deep-sea environments, efficiently degraded AFB1 while generating aflatoxicol, aflatoxin D1, and aflatoxin D2, with reduced cytotoxicity of the resulting products [53]. Under optimized conditions, the same strain achieved 88.0% detoxification within only 2 days [54]. In a food fermentation model, Rafai et al. reported that *Latilactobacillus curvatus*, *Pediococcus pentosaceus*, and *Bacillus firmus* converted AFB1 into several metabolites including aflatoxicol, aflatoxin D1, aflatoxin P2, aflatoxin Q1, and aflatoxin B2a, with the highest reduction observed for *L. curvatus* (41.1%) [55].

Food-associated fermentation systems also showed notable potential. *Lactobacillus helveticus* FAM22155 achieved 86–89% detoxification during solid-state fermentation of contaminated wheat bran and generated four degradation products (AFP1–AFP4) [56]. *Acetobacter tropicalis* AT7 and *Lactiplantibacillus plantarum* LP64, both isolated from mold-contaminated silages, reduced AFB1 by 47.8% and 57.0%, respectively [46].

Selected fungal systems were also effective. *Aspergillus niger* SF951, selected through metagenomic laccase-gene mining, reduced AFB1 by 55.67% through extracellular fractions and generated multiple transformation products [47]. In addition, *Pleurotus ostreatus* reduced AFB1 by 53–87% during mushroom cultivation on contaminated substrates [58].

Some studies reported higher detoxification activity in extracellular fractions than in intact cells. *Microbacterium proteolyticum* B204 reduced AFB1 by 77.0% in whole culture and 80.1% using the cell-free supernatant alone, suggesting that secreted proteinaceous enzymes were major active agents [57]. Similar observations were reported for *Bacillus albus*, *Burkholderia contaminans*, and *Enterococcus faecium*, further supporting the importance of extracellular detoxification mechanisms.

Collectively, the studies summarized in Table 1b indicate that microbial biodegradation systems reported substantial reductions in AFB1, frequently exceeding 80–90% and in some cases approaching near-complete removal. However, these percentages should not be interpreted as direct evidence of comparative superiority because the study designs and endpoints differed substantially. Unlike adsorption-based strategies, these approaches provide evidence of true toxin conversion and therefore represent promising platforms for advanced biological detoxification of AFB1.

### 2.3. Yeast-Based Systems Involved in AFB1 Detoxification

Yeasts have also been investigated as biological tools for AFB1 detoxification, mainly through adsorption to cell wall polysaccharides, inhibition of aflatoxigenic fungi, or direct biodegradation. Although fewer studies were identified compared with bacterial systems, several yeast species demonstrated substantial AFB1 reductions under their respective experimental conditions (Table 2).

Among the reported yeasts, *Saccharomyces cerevisiae* was the most frequently investigated species. Zolfaghari et al. observed AFB1 reductions up to 30.46% during simulated gastrointestinal digestion using *S. cerevisiae* isolated from traditional dairy products [34]. Hamad et al. reported adsorption values ranging from 52% to 99.7% depending on pH and treatment conditions, with the highest efficacy obtained using a tri-mix system combining activated charcoal, *Lacticaseibacillus rhamnosus*, and *S. cerevisiae* [64].

Engineered yeasts also showed promising performance. Huang et al. developed a recombinant *S. cerevisiae* strain expressing anti-AFB1 antibodies on the cell surface. This modified strain displayed a toxin-binding capacity 1.7-fold higher than the wild-type strain and significantly increased fecal excretion of AFB1 in vivo [65].

Some non-*Saccharomyces* species achieved AFB1 reductions above 90% under the conditions evaluated. *Geotrichum candidum* XG1 reduced AFB1 by 99.1–100% after 48 h, with transformation products detected during the process [62]. *Hanseniaspora uvarum* U1 achieved reductions between 93.7% and 99.1%, with maximum activity at pH 5.5 [63]. *Sporidiobolus pararoseus* KM281507 reached up to 93% reduction under poultry gastrointestinal simulation conditions [66].

Antagonistic activity against aflatoxigenic fungi was also observed. *Candida albicans* ATCC14053 strongly inhibited *Aspergillus parasiticus* growth in wheat grains and reduced AFB1 accumulation by 75.55% [61].

Collectively, the studies summarized in Table 2 indicate that yeasts constitute an effective complementary platform for AFB1 detoxification, particularly through cell wall adsorption and, in some strains, through active biodegradation or inhibition of aflatoxin biosynthesis.

### 2.4. Enzymatic Detoxification of AFB1

Purified enzymes and recombinant biocatalysts have been extensively investigated for direct AFB1 detoxification under controlled reaction conditions. The identified studies mainly involved laccases, dye-decolorizing peroxidases (DyPs), reductases, and other oxidative enzymes, highlighting the importance of catalytic oxidation and transformation pathways (Table 3).

Laccases were the most represented enzyme family and frequently achieved high detoxification levels. Recombinant fungal laccase rCuL reduced AFB1 by up to 94% [68]. Recombinant laccase rAnLI achieved 94.72% degradation and generated several transformation products [47]. Lac-W laccase reduced AFB1 by 88% in standard assays and up to 92% in feed matrices, with AFQ1 identified as the main metabolite [76]. Recombinant LAC3 expressed in *Saccharomyces cerevisiae* also showed high activity, reaching 90.33% detoxification [77].

CotA laccase systems showed strong catalytic activity. Recombinant BsCotA reduced AFB1 by approximately 80% within 48 h, producing AFQ1 and epi-AFQ1 [69], whereas free CotA enzyme achieved 74.4% degradation with multiple degradation products identified [70]. Immobilized laccase from *Bacillus amyloliquefaciens* further demonstrated practical applicability, reducing AFB1 by 90% in contaminated corn oil [78].

DyPs represented the second most frequent enzyme class. DypB from *Rhodococcus jostii* reduced AFB1 by 95% after 72 h [71]. BsDyP achieved reductions between 50.0% and 76.93% and generated AFB1-diol [73]. BaDyP from *Bjerkandera adusta* reduced AFB1 by 86.68% and produced AFQ1, AFB1-diol, and additional metabolites [79]. DyP from *Paracoccus* sp. XF-30 also showed relevant catalytic activity, reaching 71.63% detoxification [81].

Other catalytic systems were effective. Aldo–keto reductase MgAKR reduced AFB1 by more than 90% through conversion into aflatoxicol under NADPH-dependent conditions [67]. Lipase/protease preparations from *Humicola lanuginosa* achieved reductions ranging from 35.8% to 81.3% depending on enzyme dose [75]. Wang et al. further demonstrated that several oxidative enzymes, including laccase, DyP, lignin peroxidase, and versatile peroxidase, reduced AFB1 by 90.06–92.91%, with AFB1-8,9-dihydrodiol identified as a transformation product [80].

Not all enzymes displayed equally high efficiencies. Commercial laccase from *Trametes versicolor* reduced AFB1 by only approximately 12% after 96 h, despite the detection of ring-opened and hydroxylated products [74], indicating that enzyme origin and catalytic properties strongly influence detoxification performance.

Collectively, the studies summarized in Table 3 indicate that several enzyme-based systems achieved substantial AFB1 reductions under optimized experimental conditions. In many cases, catalytic systems achieved reductions above 90% while simultaneously generating identifiable transformation products, supporting their potential as advanced tools for future food and feed decontamination applications. Nevertheless, these results do not establish superiority over microbial systems because the experimental conditions, matrices, treatment durations, analytical methods, and measured endpoints differed among studies.

### 2.5. Experimental Application Models for AFB1 Detoxification

Several studies have evaluated biological detoxification systems in practical food, feed, and environmental matrices, demonstrating that the previously described microbial and enzymatic approaches can be translated into applied models. The investigated applications mainly included fermentation processes, grain storage protection, feed treatment, food matrix decontamination, and environmental remediation (Table 4).

Fermentation-based applications were widely represented. Escrivá et al. reported that bread making using lyophilized *Lactiplantibacillus plantarum* B3 reduced AFB1 in contaminated maize flour by 55.0% [33]. Zhang et al. observed reductions of 86–89% during solid-state fermentation of wheat bran using *Lactobacillus helveticus* FAM22155 [56]. In traditional doenjang fermentation, *Bacillus albus* YUN5 decreased total aflatoxins to 6.04 µg/kg after 12 months [48]. Additional fermentation-based detoxification was also reported for contaminated peanut meal using *Enterococcus faecium* HB2-2, with reductions ranging from 47.7% to 82.9% depending on process conditions [52].

Biocontrol during storage produced strong outcomes. *Bacillus subtilis* E11 almost completely inhibited fungal growth and significantly reduced AFB1 in dried chili [22]. *Streptomyces exfoliatus* suppressed *Aspergillus flavus* growth in wheat grains and reduced toxin levels to undetectable or trace concentrations [43]. In a similar manner, *Levilactobacillus brevis* DN-1 showed strong preservative activity in contaminated oilseed cakes, achieving complete inhibition of AFB1 production under some storage periods [31].

Feed detoxification models showed similarly promising results. *Bacillus amyloliquefaciens* YUAD7 reduced AFB1 in contaminated silage from 100 µg/kg to 1.7 µg/kg after 48 days [19]. *Sporidiobolus pararoseus* achieved 93% detoxification in poultry feed under gastrointestinal simulation conditions [66]. Lac-W laccase reduced AFB1 in contaminated corn cob by 92% [76].

Several food matrix treatments were also effective. *Microbacterium proteolyticum* B204 supernatant reduced AFB1 by 78.0% in peanuts, 83.3% in corn, and 58.7% in cheese [57]. *Geotrichum candidum* XG1 reduced AFB1 in red pepper by 83.0% [62]. Water kefir grains reduced AFB1 by 54.90–58.85% in milk and tea infusions [83]. Probiotic lactic acid bacteria preparations also reduced AFB1 in naturally contaminated cereals, reaching 52.28% in corn, 83.03% in rice, and 77.22% in wheat [82].

Digestive simulation models confirmed the potential relevance of biological detoxification under gastrointestinal conditions. Rafai et al. reported that *Latilactobacillus curvatus* 14, *Pediococcus pentosaceus* 4, and *Bacillus firmus* 6 reduced AFB1 by up to 72.3% during the colonic phase using contaminated maize flour extracts [55]. Zolfaghari et al. also reported detoxification values up to 31.14% using indigenous probiotic bacteria and yeasts during simulated digestion [34].

Environmental and industrial applications were additionally explored. Immobilized CotA laccase achieved complete removal of AFB1 from wastewater generated during washing of rice, red ginseng, and medicinal plant materials after 6 h [70]. Mycoremediation using *Pleurotus ostreatus* reduced AFB1 by 53–87% in spent mushroom substrate, while negligible toxin levels were detected in fruiting bodies [58].

Finally, incorporation into functional foods was also demonstrated. Chocolate fortification using activated charcoal, *Lacticaseibacillus rhamnosus*, and *Saccharomyces cerevisiae* reduced AFB1 by 90.2–96.8%, depending on pH and incubation conditions [64].

Collectively, the studies summarized in Table 4 indicate that biological AFB1 detoxification is not limited to laboratory buffer systems, but can be successfully applied to complex real matrices including cereals, fermented foods, feed ingredients, beverages, wastewater, and processed products. These findings support the translational potential of microbial and enzymatic detoxification strategies for practical food and feed safety management.

## 3. Discussion

The studies compiled in this review indicate that microbial detoxification of AFB1 has developed into a substantial and rapidly expanding field of research, although important disparities remain regarding the biological systems investigated, the detoxification mechanisms explored, and the extent of practical application. A first striking observation is the predominance of bacterial systems throughout the literature. Whether Table 1a,b are considered together, bacteria represent the vast majority of microbial entries, whereas only two filamentous fungal systems were identified (*Aspergillus niger* and *Pleurotus ostreatus*). This trend strongly suggests that contemporary biological detoxification research has largely prioritized bacteria because of their shorter generation times, easier cultivation, recognized food or feed relevance, probiotic potential, and broader industrial acceptability. In contrast, filamentous fungi remain comparatively underexplored despite their enzymatic richness and possible relevance as biodegradation platforms.

From a mechanistic perspective, the bacterial literature was dominated by adsorption-, binding-, and inhibition-based approaches rather than confirmed structural modification. Across Table 1a,b, approximately 62% of bacterial entries were associated with toxin adsorption, surface binding, fungal growth suppression, or inhibition of aflatoxin biosynthesis, whereas only 38% involved direct biodegradation or biotransformation. This imbalance is highly informative. It indicates that most studies still focus on preventing toxin accumulation or physically reducing toxin bioavailability rather than achieving verified molecular conversion of AFB1.

**Figure 1:**
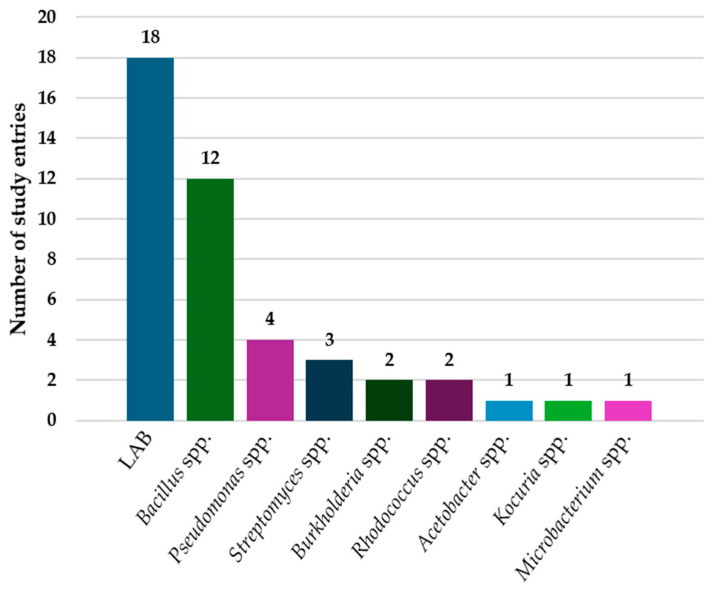
Distribution of bacterial genera/groups among bacterial study entries. Bars show raw counts. the total denominator was *n* = 44 bacterial study entries.

The taxonomic structure of the bacterial dataset was also highly concentrated. Lactic acid bacteria-related systems represented approximately 41% of all bacterial entries, whereas *Bacillus* spp. accounted for 27%. The remaining 32% included genera such as *Pseudomonas*, *Streptomyces*, *Burkholderia*, *Rhodococcus*, *Kocuria*, *Microbacterium*, and *Acetobacter*. This distribution suggests that biological detoxification research is being driven primarily by microorganisms already considered technologically relevant (Figure 1). Lactic acid bacteria dominance is not surprising, given their long history of safe use in foods, natural prevalence in fermented products, and cell-wall structures rich in peptidoglycan and polysaccharides that favor toxin adsorption. Their widespread use suggests that adsorption remains one of the most realistic strategies for immediate toxin mitigation under mild food-processing conditions.

By contrast, the recurrent presence of *Bacillus* spp. across both Table 1a,b indicates a broader functional role. *Bacillus* strains were repeatedly associated with inhibition of *Aspergillus* growth, suppression of aflatoxin biosynthesis, secretion of extracellular metabolites, and direct biodegradation of AFB1. This multifunctionality likely explains why they are the second most represented bacterial group. Additional traits such as spore formation, environmental robustness, tolerance to processing stress, and suitability for feed or agricultural applications further reinforce their industrial interest. Taken together, the dual predominance of lactic acid bacteria and *Bacillus* suggests that future microbial detoxification systems may benefit from combining rapid adsorption capacity with durable antagonistic or degradative activity.

Although less represented numerically, minority bacterial genera should not be overlooked. *Pseudomonas* and *Streptomyces* were frequently associated with potent anti-aflatoxigenic activity, while *Burkholderia*, *Rhodococcus*, *Kocuria*, and *Microbacterium* were more often linked to extracellular biodegradation or novel transformation pathways. These genera may therefore represent valuable reservoirs of enzymes or metabolites not yet fully exploited. Their lower frequency in the literature may reflect regulatory caution, limited food-grade status, or a stronger focus of previous research on probiotic organisms rather than an absence of detoxification potential [14].

The yeast literature was substantially smaller than the bacterial dataset, but still highly informative. Only seven yeast-related entries were identified, confirming that yeasts remain underrepresented in comparison with bacterial systems. Nevertheless, 43% of yeast entries involved *Saccharomyces cerevisiae*, while the remaining 57% corresponded to non-*Saccharomyces* species such as *Geotrichum candidum*, *Hanseniaspora uvarum*, *Candida albicans*, and *Sporidiobolus pararoseus*. This is an important finding because it shows that high detoxification performance is not restricted to the conventional industrial yeast *S. cerevisiae*. In fact, several non-*Saccharomyces* yeasts achieved some of the highest reductions reported in the dataset. Their performance likely reflects strong cell-wall adsorption capacity, ecological adaptation to fermented matrices, or previously underappreciated biodegradation mechanisms. This suggests that yeast biodiversity remains an underused resource for food-compatible detoxification strategies.

The enzyme dataset provided one of the strongest indications that the field is moving toward more precise catalytic detoxification systems. In Table 3, laccase-based systems represented approximately 56% of all enzyme entries, whereas dye-decolorizing peroxidases (DyPs) accounted for 25%. Overall, oxidative enzymes constituted nearly 88% of the enzyme dataset, demonstrating that oxidation-driven transformation is currently the dominant catalytic paradigm for AFB1 detoxification. This is mechanistically coherent, since oxidative enzymes can target reactive moieties of AFB1 and convert the toxin into derivatives such as AFQ1, AFB1-diol, or related metabolites [17]. Importantly, 50% of enzyme entries reported detoxification values of 90% or higher, showing that substantial AFB1 reductions can be achieved under specific optimized reaction conditions.

However, enzyme systems also highlight a recurrent challenge in the field: high efficacy under controlled laboratory conditions does not automatically guarantee industrial feasibility. Many enzyme assays were performed in buffer systems, at optimized pH values, controlled temperatures, purified toxin concentrations, or with added cofactors. Such conditions are ideal for mechanistic demonstration but may differ considerably from real food and feed environments. Factors such as matrix complexity, enzyme instability, cofactor cost, process integration, and regulatory approval remain substantial barriers to implementation. Thus, while enzyme-based detoxification appears highly promising, its transition from laboratory success to commercial reality still requires significant development [84,85].

Studies regarding practical application and summarized in Table 4 further illustrate this translational gap. A total of 21 applied models were identified, but they were unevenly distributed. Fermentation-related systems represented approximately 38% of application entries, making fermentation the most common practical context. By contrast, direct food-oriented applications in the strict sense represented a much smaller proportion. Even when including bread making, sourdough, chocolate fortification, red pepper treatment, milk/tea co-incubation models, and post-harvest cereal treatments, the number of studies directly addressing consumer food products remained limited relative to the overall literature.

Gastrointestinal simulation models accounted for around 14%, while several other studies focused on silage, feed detoxification, grain storage, or wastewater treatment. This indicates that many biological detoxification studies still prioritize proof-of-concept or supportive matrices rather than commercial food processing conditions. This point is particularly important because although numerous studies reported high detoxification percentages, only a limited number evaluated systems in real foods where sensory quality, texture, flavor, nutrient retention, shelf life, process timing, and consumer acceptance become critical constraints. A bacterial strain that performs well in phosphate buffer may behave very differently in cheese, bread dough, spices, nuts, or oil-rich matrices. Similarly, detoxification observed during gastrointestinal simulation is valuable for assessing post-ingestion toxin binding, but it does not solve contamination at the production stage [11,86].

The toxicological evaluation of AFB1 degradation products also remains an important limitation. A reduction in the concentration of the parent toxin or the identification of transformation products does not necessarily demonstrate that the resulting compounds are less toxic. Although reduced cytotoxicity was reported for the products generated by *Kocuria rosea* [53], most studies did not directly assess cytotoxicity, genotoxicity, mutagenicity, or in vivo toxicity. Therefore, further toxicological characterization of degradation products is required before these biological strategies can be considered safe for practical food and feed applications.

In addition to detoxification performance, biosafety should be considered when selecting microbial or enzymatic systems for food and feed applications. Food-associated microorganisms may offer practical advantages; nevertheless, their suitability should be confirmed at the strain level, including accurate identification and evaluation of potential virulence, toxigenicity, antimicrobial-resistance determinants, and undesirable metabolite production. Environmental isolates and microorganisms without an established history of safe use may require more extensive assessment. For enzymatic approaches, the safety of the production organism, enzyme preparation, and any residual materials in the treated matrix should also be considered before practical implementation.

Overall, the evidence collected in this review indicates that biological AFB1 detoxification has progressed from exploratory screening toward mechanistically informed biotechnological development. Bacteria dominate the current literature, particularly lactic acid bacteria and *Bacillus* spp., yeasts remain promising but underexplored, oxidative enzymes offer high catalytic efficiency, and practical applications are increasing but still limited. The next decisive step for the field is no longer discovering whether microorganisms can detoxify AFB1, but determining which systems can do so safely, reproducibly, and at industrial scale. Future advances will likely emerge from integrated strategies combining adsorption, fungal inhibition, catalytic degradation, and real-matrix validation within standardized process frameworks.

## 4. Conclusions

This review highlights the considerable progress achieved in the biological detoxification of AFB1 and confirms that microorganisms and enzymes can effectively reduce toxin levels through multiple complementary mechanisms. Bacterial systems, particularly lactic acid bacteria and *Bacillus* species, dominate the current literature and mainly operate through adsorption, inhibition of fungal growth, and suppression of aflatoxin biosynthesis. At the same time, biodegradation and biotransformation approaches, although less frequently reported, provide strong evidence of structural modification and the formation transformation products; however, reduced toxicity was directly demonstrated in only a limited number of studies.

Yeasts have shown notable detoxification capacities despite being less extensively studied, especially among non-*Saccharomyces* species, suggesting that microbial biodiversity remains an underexplored resource. Enzymatic systems, particularly oxidative enzymes such as laccases and dye decolorizing peroxidases, frequently reported AFB1 reductions exceeding 90% under optimized conditions. These findings indicate that catalytic oxidation represents a key pathway for effective AFB1 transformation. However, these values are study-specific and should not be interpreted as direct evidence of superiority because of the substantial methodological heterogeneity among the included studies.

An important gap remains between laboratory scale efficiency and real-world applicability. Most studies were performed under simplified experimental conditions, while relatively few addressed detoxification in complex food and feed matrices. Factors such as matrix composition, sensory quality, regulatory constraints, and process scalability continue to limit industrial implementation. In addition, the safety of degradation products and the reproducibility of results under realistic conditions require further investigation.

Overall, biological detoxification represents a promising and sustainable strategy for AFB1 mitigation. Future research should focus on integrating highly efficient microorganisms or enzymes into practical food and feed systems, optimizing process conditions, and ensuring the safety and regulatory acceptance of the resulting products. Bridging the gap between experimental performance and industrial feasibility will be essential for translating these advances into effective large scale applications.

## 5. Materials and Methods

### 5.1. Search Strategy and Eligibility Criteria

The methodological rigor of this systematic review, as well as the minimization of potential bias, was ensured through compliance with the Preferred Reporting Items for Systematic Reviews and Meta-Analyses (PRISMA) guidelines for the identification, screening, and selection of relevant studies [87]. The completed PRISMA 2020 Checklist and PRISMA 2020 for Abstracts Checklist are provided as Supplementary Files S1 and S2, respectively. The protocol of this systematic review was not registered in a public registry such as PROSPERO, the Open Science Framework, or INPLASY. A comprehensive literature search was conducted using three major electronic databases: PubMed, Web of Science, and Scopus. The search covered the period from January 2020 to December 2025 in order to capture the most recent scientific evidence related to the biological detoxification of AFB1.

The search strategy was developed using the following keywords and Boolean operators: (“Aflatoxin B1” OR “AFB1”) AND (“biological detoxification” OR “biotransformation” OR “binding”) AND (“bacteria” OR “enzyme” OR “fungi” OR “probiotic”). In PubMed, this search strategy yielded records that were sufficiently specific and directly relevant to the objectives of the review, and therefore no additional refinement was required. In contrast, the broader search outputs obtained from Web of Science and Scopus required a subsequent refinement step using the Boolean combination (“detoxification” OR “decontamination”) AND “biological” AND “aflatoxin B1”, thereby improving the specificity of the retrieved studies and ensuring their relevance to the review topic.

Only original research articles published in English and specifically focused on biological approaches for AFB1 detoxification were considered eligible for inclusion. Review articles, conference abstracts, studies unrelated to AFB1, and publications addressing exclusively chemical or physical detoxification strategies were excluded.

### 5.2. Systematic Review Process

The literature search identified a total of 220 records across the three selected databases, including 75 records retrieved from PubMed. In Web of Science and Scopus, the initial broad searches generated 373,257 and 234 records, respectively, following refinement, these results were reduced to 64 and 81 relevant studies, respectively.

After removal of 45 duplicate records, 175 studies remained for title and abstract screening. During this stage, 37 records were excluded because they did not meet the scope or objectives of the present review. Consequently, 138 full-text articles were assessed for eligibility.

At the full-text evaluation stage, 71 studies were excluded for the following reasons: insufficient methodological information or inaccessible full texts (n = 8), primary focus on preventive effects of biomolecules rather than direct detoxification mechanisms (n = 9), or emphasis on chemical and phytochemical detoxification strategies instead of biological approaches (n = 54).

**Figure 2:**
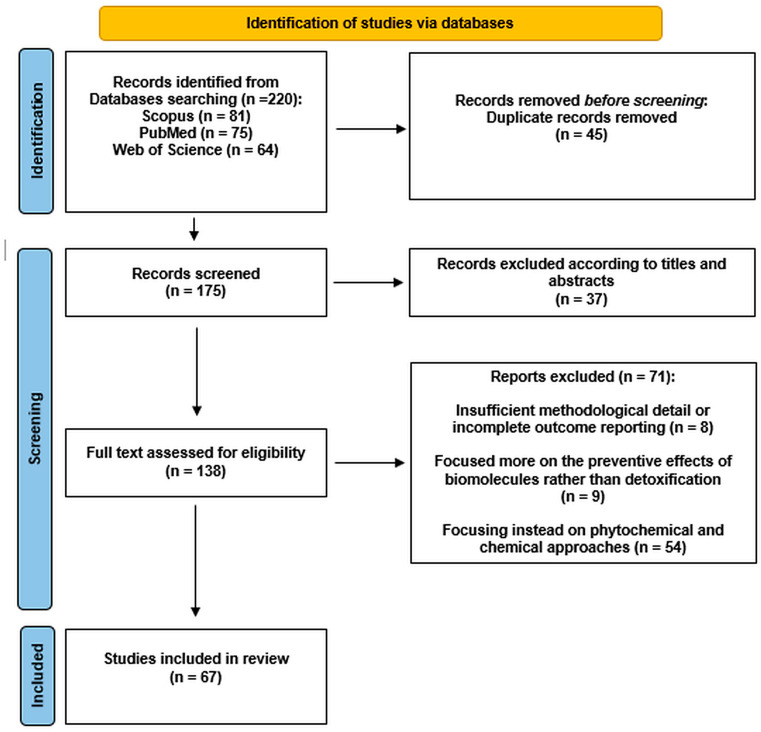
PRISMA flowchart for the total number of articles identified (n = 220) in the literature research (2020–2025).

Ultimately, 67 studies fulfilled all inclusion criteria and were retained for qualitative synthesis in the present systematic review (Figure 2). The selected studies were subsequently categorized according to the principal detoxification mechanisms investigated, including microbial biodegradation, adsorption/binding processes, enzymatic detoxification, and applications in food and feed matrices. The present systematic review specifically focused on microbial biological detoxification, including bacteria, yeasts, and microbial enzymes, because recent literature increasingly distinguishes these approaches from chemical strategies such as plant extracts, organic acids, synthetic reagents, and other non-microbial intervention platforms. This distinction is particularly relevant since biological systems rely on adsorption, enzymatic conversion, competitive exclusion, or metabolic transformation, whereas phytochemical or chemical approaches mainly depend on the chemical structure of the compounds, which determines their reactivity, mechanism of action, safety profile, and industrial applicability. Accordingly, restricting the scope to microbial systems enabled a clearer evaluation of true biological detoxification platforms and their technological potential [11]. Studies reporting more than one detoxification mechanism or both mechanistic and application-related outcomes could be included in more than one table; however, each publication was counted only once in the total number of included studies.

For the purposes of this review, adsorption or binding was defined as the physical association of AFB1 with microbial cells, cell-wall components, or other biological materials without demonstrated chemical modification of the toxin. Inhibition of toxin production referred to the suppression of fungal growth, aflatoxin biosynthesis, or both. Biodegradation was defined as a biologically mediated breakdown of AFB1 supported by evidence beyond simple adsorption, whereas biotransformation referred to the conversion of AFB1 into structurally modified products. The term confirmed detoxification was reserved for studies demonstrating AFB1 removal or transformation together with an experimentally verified reduction in the toxicity or biological activity of the treated material or resulting products.

### 5.3. Bacterial Nomenclature Standardization

Bacterial nomenclature was standardized according to currently accepted taxonomic names. For species affected by the 2020 reclassification of the genus *Lactobacillus*, the updated genus names are used throughout the manuscript. Names appearing in article titles in the reference list were not modified.

## Figures and Tables

**Table 1:** (**a**) Bacteria involved in biological detoxification by binding or inhibition of AFB1 production: strains, sources, culture conditions, and reduction efficiencies. Bacterial names were standardized using currently accepted nomenclature. (**b**) Microbial systems involved in AFB1 biodegradation and biotransformation: strains, sources, culture conditions, and reduction efficiencies. Bacterial names were standardized using currently accepted nomenclature.

(**a**)							
**Organism (Strain)**	**Source/Origin**	**Experimental Conditions**	**Key Results**	**Mechanism of Detoxification**	**Metabolites Detected**	**Analytical Quantification Method**	**Reference**
*Bacillus amyloliquefaciens* WF2020	Naturally fermented pickles	LB liquid culture with AFB1 (1–8 µg/mL), 37–45 °C, 72–96 h	Complete inhibition of AFB1 production in co-culture	Inhibition of *Aspergillus flavus* growth and aflatoxin biosynthesis	-	HPLC; HPLC–QTOF-MS	(Chen et al., 2022) [18]
*Bacillus amyloliquefaciens* YUAD7	Yak manure	Co-culture in PDB with *Aspergillus flavus* conidia (1 × 10^8^ conidia/mL) and bacterial cells (1 × 10^7^ CFU/mL), 30 °C, 48 h, 200 rpm	Significant inhibition of *A. flavus* growth and total inhibition of AFB1 production compared with control	Inhibition of *A. flavus* growth and AFB1 biosynthesis	-	UPLC–Q-TOF/MS	(Tang et al., 2024) [19]
*Bacillus amyloliquefaciens* YUAD7	Yak manure, Qinghai–Tibet Plateau	Liquid culture with AFB1 (10 µg/mL), 37 °C, 72 h	AFB1 detoxification reached 91.7% in liquid medium and >85% in food matrices	Binding and degradation via extracellular excretions	C_12_H_14_O_4_, C_5_H_12_N_2_O_2_, C_10_H_14_O_2_, C_4_H_12_N_2_O	UPLC-Q-Orbitrap HRMS, NMR	(Tang et al., 2024) [20]
*Bacillus* sp. MA82	Broiler gastrointestinal tract and fecal samples	Whole-cell binding assay in PBS with AFB1 (5 µg/mL), 37 °C, 0–24 h	AFB1 adsorption increased from 45% at 0 h to 75% after 24 h	Cell wall adsorption/binding	-	HPLC	(Abdolmaleki et al., 2025) [21]
*Bacillus subtilis* (E11; compared with V1J1 and 9932)	Isolated from fermented foods (China)	In vitro confrontation with *A. flavus*; liquid culture assays in LB, PDA, and PDB media, 28–37 °C, 24–120 h	Growth inhibition of *A. flavus* (~64%); AFB1 removal up to 81.34% after 24 h	Inhibition of fungal growth; inhibition of AFB1 biosynthesis	-	ELISA	(Yuan et al., 2023) [22]
*Bacillus subtilis* RWGB1, *Bacillus oceanisediminis* RWGB2, *Bacillus firmus* RWGB3, *Pseudomonas aeruginosa* RWGB4	Rice weevil gut	In vitro incubation with AFB1, 30 °C, 72 h	AFB1 detoxification ranged from 48.9% to 84.2%, depending on strain	Binding to AFB1	-	LC-MS	(Al-Saadi et al., 2024) [23]
*Bacillus thuringiensis* AMK10/1; *Lysinibacillus boronitolerans* AMK9/1; *Lysinibacillus fusiformis* AMK10/2; *Rummeliibacillus suwonensis* AMK9/2	Fermented forages (Hungary)	PBS system with AFB1 (24 µg/L), viable cells or cell wall fractions, 25 °C, 1 h	AFB1 elimination was generally <20%; maximum removal reached 64% with the S-layer fraction of *B. thuringiensis* AMK10/1	Cell wall adsorption mediated by S-layer proteins	-	HPLC-FLD	(Adácsi et al., 2022) [24]
*Enterococcus faecium* (Lab-L4/al), *Enterococcus durans* (Lab-L1), *Lactiplantibacillus plantarum* R1096	Algerian fermented wheat (El-Hammoum) and fermented milk	CPB buffer with AFB1 (40 ng/mL), pH 5–6, 10^10^ CFU/mL, viable or heat-inactivated cells, 25 °C, 24 h	AFB1 adsorption ranged from 25% to 80%, with highest activity observed for nonviable cells	Cell wall adsorption	-	HPLC-FLD	(Badji et al., 2023) [25]
*Lactiplantibacillus pentosus TV3*	Animal fecal samples	Cell suspension (10^8^ CFU/mL) with AFB1, 37 °C, 10 min	AFB1 adsorption reached 11.5%	Binding- Cell wall adsorption	-	HPLC-UV	(Kosztik et al., 2020) [26]
Lactic acid bacteria	probiotic dairy foods (fermented milk drinks, probiotic yogurt)	Incubation with AFB1, pH 5.5, 37 °C, 72 h	AFB1 detoxification reached 46% with live cells and 62% with denatured cells	Adsorption to bacterial cell walls	AFB1-8,9-dihydrodiol	LC-MS/MS	(Ondiek et al., 2022) [27]
Lactic acid bacteria	Dairy and food-associated lactic acid bacteria collections	Co-culture with *Aspergillus parasiticus* NRRL 2999 in YES medium, 25 °C, 7 days; adsorption assay in phosphate buffer with AFB1 (1 µg/mL)	Inhibition of AFB1 production reached 100% depending on strain; AFB1 adsorption ranged from 40% to 70%	Inhibition of fungal growth and aflatoxin biosynthesis; cell wall adsorption	-	HPLC–FLD	(Møller et al., 2021) [28]
*Lacticaseibacillus paracasei* subsp. *paracasei* CCMA 1764; *Levilactobacillus brevis* CCMA 1762; *Lactiplantibacillus pentosus* CCMA 1768	Naturally fermented Brazilian table olives	Dual-culture overlay assay and cell-free supernatant assay against *Aspergillus flavus*; CFS tested at 300–500 µL/mL, 25 °C, 7 days; additional MRS broth assays for 12 days	Complete inhibition of *A. flavus* growth; 100% AFB1 detoxification at 500 µL/mL CFS; AFB1 adsorption reached 48–51%	Growth inhibition of *Aspergillus* spp.; cell wall adsorption	Aflatoxin B2a	HPLC–FLD for AFB1 quantification UHPLC for AFB2a confirmation	(Simões et al., 2023) [29]
*Lactobacillus acidophilus* ATCC 4356 *Lacticaseibacillus casei* ATCC 39392	Probiotic reference strain	Simulated gastrointestinal model with AFB1 (5 µg/mL); oral, gastric (pH 2.5), and intestinal (pH 7.5) phases, ~4 h	AFB1 detoxification ranged from 24.3% to 70.0%	Cell wall adsorption	-	HPLC–FLD	(Tajik and Sayadi, 2020) [30]
*Levilactobacillus brevis* DN-1	Moldy feed samples	MRS medium with AFB1 (5 µg/L), anaerobic incubation, 37 °C, 24–48 h	AFB1 reduction 71.38% in liquid culture	Cell wall adsorption; inhibition of *Aspergillus* growth and AFB1 biosynthesis	-	HPLC-FLD	(Wang et al., 2024) [31]
*Lactiplantibacillus plantarum* ATCC 8014 *Lacticaseibacillus rhamnosus* ATCC 7469	Iranian Research Organization for Science and Technology	AFB1-contaminated sourdough (10 µg/kg), thermosonicated co-culture, 27–37 °C, 8–24 h	Maximum AFB1 adsorption reached 8.04 µg/kg under thermosonicated co-culture conditions (37 °C, 24 h)	Cell wall adsorption mainly driven by hydrophobic interactions	-	HPLC-FLD	(Abedi et al., 2022) [32]
*Lactiplantibacillus plantarum B3, Lacticaseibacillus paracasei B10*	Goat milk whey	MRS broth with AFB1, 37 °C, 72 h	AFB1 detoxification ranged from 27% to 55%	Cell wall adsorption		HPLC-QTOF-MS	(Escrivá et al., 2023) [33]
*Lacticaseibacillus rhamnosus*	Traditional dairy products	Simulated gastrointestinal model with AFB1 (10 ppb), 1 × 10^10^ CFU/mL, gastric phase pH 2.5, intestinal phase pH 7.5, 37 °C, 4 h	AFB1 detoxification reached 31.14%	Cell wall adsorption	-	ELISA	(Zolfaghari et al., 2020) [34]
*Lacticaseibacillus rhamnosus* GG	Commercial probiotic	PBS system with AFB1 (1 µg/mL), 2 × 10^9^ CFU, viable or heat-treated cells, NaCl 0–1.8%, 25–37 °C, 1–2 h	Maximum AFB1 adsorption reached 97.74% with heat-treated cells (1% NaCl, 1 h); desorption remained ≤3% after in vitro digestion	Non-covalent cell wall adsorption	-	HPLC–FLD	(Balsini et al., 2021) [35]
*Levilactobacillus brevis, Lactobacillus helveticus, Lactoplantibacillus plantarum, Leuconostoc pseudomesenteroides, Weissella confusa, Weissella cibaria*	Food-derived strains (maize porridge, dairy products)	Incubation with AFB1 at pH 3 or pH 7, 25–37 °C, 24–48 h	AFB1 detoxification ranged from 16% to 71%, depending on strain	Surface adsorption to lactic acid bacteria cells	-	LC-QTOF-MS, UPLC-FLD	(Lemmetty et al., 2025) [36]
*Limosilactobacillus fermentum*	Fermented coconut toddy (“tuba”), Philippines	Incubation with AFB1 (1.64 ppb), live or heat-treated cells, 37 °C, 1 h	AFB1 adsorption reached 87.30 ± 7.29% with live cells and 70.49 ± 9.59% with heat-treated cells	Cell wall adsorption	-	ELISA	(Baltazar et al., 2025) [37]
*Pediococcus acidilactici* OR83	Animal fecal samples	Cell suspension (10^8^ CFU/mL) with AFB1, 37 °C, 10 min	AFB1 adsorption reached 7.6%	Cell wall adsorption	-	HPLC-UV	(Bata-Vidács et al., 2020) [38]
*Pediococcus pentosaceus* (N17-02, N19-37, N57-15, N57-24) *Weissella paramesenteroides* (N33-01, N44-02); *Companilactobacillus crustorum* RL48-10	Korean Nuruk (fermentation starter)	Transwell co-culture with *Aspergillus flavus*, 25 °C, 3–8 days	Significant inhibition of AFB1 production	Inhibition of AFB1 biosynthesis; cell wall adsorption; production of antifungal metabolites	Lactic acid, 4-hydroxybenzaldehyde, adenine, 2,3-cAMP	LC-QTOF-MS, LC-MS/MS	(Yun et al., 2024) [39]
*Pediococcus pentosaceus* L6; *Lactiplantibacillus plantarum* L12; *Leuconostoc mesenteroides* L18, L19; *Loigolactobacillus coryniformis* subsp. *coryniformis* L47; *Levilactobacillus brevis* L52	Brewer’s grains (Argentina)	PBS system with AFB1 (150 ng/mL), viable or heat-treated cells, 37 °C, 4 h	AFB1 adsorption ranged from 37.6% to 70.7%	Cell wall adsorption	-	HPLC-FLD	(Asurmendi et al., 2020) [40]
*Pseudomonas* (Ps-4, 66, 68)	Corn rhizosphere	Solid co-culture with *Aspergillus flavus*, 25 °C, 7 days; liquid co-culture, 28 °C, 3 days, 130 rpm	Complete inhibition of AFB1 production (>99%)	Inhibition of AFB1 biosynthesis	-	HPLC-HRMS	(Papp et al., 2024) [41]
*Rhodococcus turbidus PD630*	Soil	Co-culture system with AFB1, 30 °C, 72 h	AFB1 detoxification reached 93.04% after 72 h	Cell wall adsorption	-	HPLC, UV	(Liu et al., 2023) [42]
*Streptomyces exfoliatus Agricultural soils (Egypt)*	Agricultural soils (Egypt)	Cell-free culture filtrate produced in starch nitrate broth, 30 °C, 7 days; tested against *Aspergillus flavus* and AFB1	Complete inhibition of AFB1 production at ≥20% (*v*/*v*) culture filtrate	Inhibition of fungal growth and sporulation; inhibition of aflatoxin B1 biosynthesis	-	Thin-layer chromatography with fluorodensitometric quantification	(El-Shanshoury et al., 2022) [43]
*Streptomyces* spp. (59 soil isolates including IX20, IX45) *and Streptomyces griseoviridis (Mycostop^®^)*	Organic amendments and soil samples	Dual culture with *Aspergillus flavus* and AFB1 degradation assays in solid (CYA) and liquid (CYB) media, 25 °C	Strong inhibition of *A. flavus* growth; marked reduction in AFB1 accumulation; residual AFB1 decreased to 6% for selected isolates	Inhibition of fungal growth and AFB1 biosynthesis	-	HPLC–MS	(Campos-Avelar et al., 2021) [44]
*Weissella confusa* KR780676	Indian traditional fermented food	Exopolysaccharide (EPS) extracted from MRS broth, 30 °C, 48 h	AFB1 detoxification reached 32.40% at 50 mg/mL and 34.79% at 100 mg/mL	Binding to galactan exopolysaccharide	-	HPTLC, PSA	(Kavitake et al., 2020) [45]
(**b**)							
**Organism (Strain)**	**Source/Origin**	**Experimental Conditions**	**Key Results**	**Mechanism of Detoxification**	**Metabolites Detected**	**Analytical Quantification Method**	**Reference**
*Acetobacter tropicalis* AT7/and *Lactiplantibacillus plantarum* LP64	Mold-contaminated silages	Incubation with AFB1 (50 µg/L), 37 °C, 72 h	AFB1 detoxification reached 47.8% and 57.0%, respectively	Biodegradation	-	UPLC–MS/MS	(Bao et al., 2024) [46]
*Aspergillus niger* SF951	Cultured microbiome associated with *Salvia miltiorrhiza* (strain screened by metagenomic laccase-gene mining)	Liquid incubation with AFB1 (0.5 µg/mL), 37 °C, 3 days	AFB1 detoxification reached 55.67%	Biodegradation mainly by extracellular fractions	C_16_H_22_O_4_, C_16_H_35_O_2_N, C_24_H_30_O_6_, C_18_H_39_O_2_N	HPLC–MS (AFB1 quantification); UHPLC–MS/MS	(Zhao et al., 2025) [47]
*Bacillus albus* YUN5	Doenjang (Korean fermented soybean paste)	Cell-free supernatant with AFB1 (100 ng/mL), 35 °C, 7 days	AFB1 detoxification reached 76.28%	Biodegradation mediated by non-proteinaceous metabolites present in the supernatant	Six degradation products (m/z 316, 286, 361, 317, 302, 244)	HPLC-FLD; LC–MS	(Kumar et al., 2023) [48]
*Bacillus amyloliquefaciens* WF2020	Naturally fermented pickles	LB liquid culture with AFB1 (1–8 µg/mL), 37–45 °C, 72–96 h	AFB1 detoxification exceeded 80% after 72 h	Extracellular enzyme-mediated biodegradation	C_15_H_11_O, C_15_H_15_O_2_, C_15_H_19_O_4_	HPLC; HPLC–QTOF-MS	(Chen et al., 2022) [18]
*Bacillus amyloliquefaciens* YUAD7	Yak manure	Artificially contaminated alfalfa silage with AFB1 (100 µg/kg), inoculated at 10^8^ CFU/mL, 20 °C, 48 days	AFB1 detoxification reached 99.7%, with a final concentration of 1.7 µg/kg	Biodegradation	C_12_H_14_O_4_, C_5_H_12_N_2_O_2_, C_10_H_14_O_2_, C_4_H_12_N_2_O,	UPLC–Q-TOF/MS	(Tang et al., 2024) [19]
*Bacillus licheniformis* QT338 (strain S51)	Chicken intestine	Liquid culture with AFB1 (100 ng/mL), 30 °C, 72 h	AFB1 detoxification reached 61.03%	Biodegradation	-	ELISA	(Dong et al., 2024) [49]
*Burkholderia contaminans* BC11-1	Forest rhizosphere soil, Luzhou, China	Incubation at 28 °C, 7 days, physically separated from AFB1 by a 0.22 µm membrane filter	AFB1 detoxification reached 90% without direct contact	Extracellular metabolite-mediated biodegradation	-	ELISA	(Hua et al., 2024) [50]
*Burkholderia* sp. XHY-12	Corn soil	Liquid fermentation with AFB1 (2.5 µg/mL), 37 °C, 60 h	AFB1 detoxification reached 85.2%	Biodegradation by extracellular enzymes	-	HPLC	(Yang et al., 2020) [51]
*Enterococcus faecium* HB2-2	Soil (China)	Nutrient broth with AFB1 under optimized alkaline conditions, 32 °C, up to 96 h	AFB1 detoxification reached 90.0%; fermentation supernatant showed highest activity	Biodegradation mediated mainly by extracellular proteinaceous components	Degradation products with(m/z 331, 287, and 249)	HPLC with fluorescence detection; LC–MS	(Feng et al., 2024) [52]
*Kocuria rosea* (strain 13)	Deep-sea origin	Liquid culture in M2 medium; AFB1 incubation up to 5 days	Efficient AFB1 degradation with reduced cytotoxicity of degradation products	Biotransformation	Degradation products including aflatoxicol, aflatoxin D1 and aflatoxin D2	HPLC–HRMS	(Wang et al., 2024) [53]
*Kocuria rosea* strain 13	Deep-sea environment (West Pacific Ocean)	Liquid culture with AFB1; optimized conditions: 30 °C, 2 days, pH 7.11, seawater 100%	AFB1 detoxification reached 88.0%	Biodegradation	-	HPLC-UV	(Wang et al., 2023) [54]
*Latilactobacillus curvatus* 14; *Pediococcus pentosaceus* 4; *Bacillus firmus* 6	Culture collection (CECT, Spain)	Fermentation of contaminated maize flour extract, 30–37 °C, 12–48 h	AFB1 reduction up to 41.1% (L. curvatus 14), 25.4% (*P. pentosaceus* 4), 25.1% (B. firmus 6)	Biotransformation	Aflatoxicol; aflatoxin D1; aflatoxin P2; aflatoxin Q1; aflatoxin B2a	LC–QTOF–MS	(Rafai et al., 2025) [55]
*Lactobacillus helveticus* FAM22155	Laboratory culture collection (China)	Solid-state fermentation of wheat bran contaminated with AFB1, 37 °C, 48 h, compared with liquid fermentation	AFB1 detoxification reached 86–89% during solid-state fermentation	Protein-mediated biotransformation during solid-state fermentation	AFP1, AFP2, AFP3 and AFP4	ELISA for aflatoxin B1 quantification; UHPLC–QTOF–MS for degradation product analysis	(Zhang et al., 2021) [56]
*Microbacterium proteolyticum* B204	Bovine faeces	Liquid culture with AFB1 (10 µg/mL), 30 °C, 24 h; whole culture and cell-free supernatant evaluated	AFB1 detoxification reached 77.0% in whole culture and 80.1% in supernatant	Extracellular proteinaceous enzyme-mediated biodegradation	-	HPLC-FLD	(Yan et al., 2022) [57]
*Pleurotus ostreatus*	Commercial mushroom strain	Cultivation on contaminated substrate (commercial substrate + maize, 1:1), 25 °C, 3 weeks of mycelial growth	AFB1 detoxification ranged from 53% to 87%	Biodegradation	-	HPLC–FLD	(Zapaśnik et al., 2025) [58]
*Pseudomonas fluorescens* (SZ1)	Soil, (Egypt)	Nutrient broth supplemented with AFB1 (100 ppb), 37 °C, 72 h	AFB1 detoxification reached up to 99%	Extracellular proteinaceous component-mediated biodegradation	-	HPLC with fluorescence detection	(Ali et al., 2021) [59]
*Pseudomonas knackmussii* AD02	Peanut-growing soil (Thailand)	Nutrient broth with AFB1 (100 ng/mL), pH 7.0, 25 °C, 24 h	AFB1 detoxification ranged from 88.85% to 90.0% across 20–500 ng/mL	Extracellular enzyme-mediated biodegradation	-	HPLC-FLD	(Maneeboon et al., 2024) [60]

**Table 2:** Yeasts involved in biological detoxification of aflatoxin B1: strains, sources, culture conditions, and reduction efficiencies.

Organism (Strain)	Source/Origin	Experimental Conditions	Key Results	Mechanism of Detoxification	Metabolites Detected	Analytical Quantification Method	Reference
*Candida albicans* ATCC14053	-	Wheat grains asynchronously inoculated with yeast (10^7^ CFU/mL) and *Aspergillus parasiticus* spores (10^4^ spores/g), 28–30 °C, 12 days	Strong inhibition of *A. parasiticus* growth; AFB1 detoxification reached 75.55% in wheat grains	Inhibition of AFB1 biosynthesis	-	HPLC-FLD	(Aghamohseni et al., 2022) [61]
*Geotrichum candidum* XG1	Traditional Chinese fermented foods	Liquid culture with AFB1, 30 °C, 48 h	AFB1 detoxification reached 99.1–100%	Biodegradation	C_17_H_14_O_8_, C_17_H_16_O_8_	HPLC-FLD; UPLC-QTOF-MS/MS	(Yang et al., 2024) [62]
*Hanseniaspora uvarum* U1	Spanish grape berries	PDB medium with AFB1 (1 µg/L), viable or heat-inactivated cells, pH 3.0–7.0, 30 °C, 48 h	AFB1 detoxification ranged from 93.7% to 99.1%, with maximum activity at pH 5.5	Cell wall adsorption; possible active degradation	-	ELISA	(Gómez-Albarrán et al., 2021) [63]
*Saccharomyces cerevisiae*	Traditional dairy products	Simulated gastrointestinal model with AFB1 (10 ppb), 2 × 10^8^ cells/mL, 37 °C	AFB1 detoxification reached 30.46%	Cell wall adsorption	-	ELISA	(Zolfaghari et al., 2020) [34]
*Saccharomyces cerevisiae*	Commercial strains	In vitro PBS model simulating gastrointestinal conditions, pH 3.0 and 6.8, 2–4 h	AFB1 adsorption ranged from 52% to 99.7%, with highest activity in tri-mix formulation	Cell wall adsorption	-	HPLC	(Hamad et al., 2023) [64]
*Saccharomyces cerevisiae*-pYD1-ScFv-AFB1 (engineered strain)	Engineered from *S. cerevisiae* ATCC 9763	In vitro binding assay, in vivo mouse exposure model with AFB1 (0.3 mg/kg/day) for 4 weeks; oral administration of 1 × 10^9^ CFU/day	AFB1 binding capacity was 1.7-fold higher than wild-type yeast, fecal AFB1 excretion increased	Specific bio-binding mediated by surface-displayed anti-AFB1 single-chain antibody	-	HPLC (feces)	(Huang et al., 2025) [65]
*Sporidiobolus pararoseus* KM281507	Red yeast cells (spray-dried, encapsulated)	Incubation with AFB1 (1–5 µg/mL), 25–37 °C, 48 h, anaerobic conditions	AFB1 detoxification reached up to 93% at low dose under poultry gastrointestinal model conditions	Cell wall adsorption mediated by β-glucan-rich biomass	-	ELISA, HPLC-FLD	(Tapingkae et al., 2022) [66]

**Table 3:** Enzymes catalyzing aflatoxin B1 degradation: sources, reaction conditions, incubation times, and reduction efficiencies.

Enzyme Name	Source/Origin	Reaction Conditions	Time/Dose	% AFB1 Reduction	Detected Metabolites	Analytical Method	Reference
Aldo–keto reductase (MgAKR, gene MG2-4)	*Meyerozyma guilliermondii* AF01	In vitro phosphate buffer system (pH 5.0–7.0), 30–37 °C, NADPH-dependent	1350–1620 µg/mL; NADPH up to 4.8 mM, 72 h	AFB1 detoxification exceeded 90% under optimal conditions	Aflatoxicol	LC–MS	(Zhang et al., 2025) [67]
Recombinant fungal laccase (rCuL)	*Cerrena unicolor* 6884	Buffer system at pH 7.0–8.0, 45–65 °C, initial AFB1 concentration 2.0 µg/mL	1 U/mL purified enzyme, 24 h	AFB1 detoxification reached 94%	-		(Zhou et al., 2022) [68]
Recombinant laccase rAnLI	Laccase gene AnLI from *A. niger* SF951 purified enzyme	pH 5.0, 35 °C, 1 mM Cu^2+^, AFB1 (1 µg/mL)	0.1 µg/mL enzyme, 48 h	AFB1 detoxification reached 94.72%	C_16_H_22_O_4_, C_16_H_35_O_2_N, C_24_H_30_O_6_, C_18_H_39_O_2_N	UHPLC–MS/MS	(Zhao et al., 2025) [47]
CotA laccase (BsCotA, recombinant)	*Bacillus subtilis* spore coat protein	100 mM phosphate buffer (pH 7.0), 37 °C, Cu^2+^ incorporated, no mediator	0.2 µM, 48–72 h	AFB1 detoxification reached ~80% within 48 h	AFQ1, epi-AFQ1	LC-MS/MS	(Subagia et al., 2024) [69]
CotA laccase (free form)	*Bacillus subtilis* (recombinant expression in *E. coli*)	PBS buffer (pH 7.4–8.0), 50–70 °C	20 µg, 1–4 h	AFB1 detoxification reached ~74.4%	AFQ1; AP347; AP331; AP317; AP301; AP259; AP235; AP223; AP155; AP141	UPLC–QTOF–MS	(Zhang et al., 2025) [70]
DypB (WT)	*Rhodococcus jostii;* recombinant expression in *E. coli*	Sodium malonate buffer (pH 6.0), 0.1 mM H_2_O_2_, 2 mM Mn^2+^, 25 °C	0.1 U/mL enzyme, up to 72 h	AFB1 detoxification reached 95 after 72 h	C_17_H_14_O_6_, C_16_H_14_O_6_, C_16_H_14_O_7_, C_17_H_14_O_7_	LC–HRMS	(Mangini et al., 2024) [71]
Laccase frL103 (wild type; T418A, T418S)	*Bacillus vallismortis*; recombinant expression in *E. coli*	Tris–HCl buffer (50 mM, pH 7.0), 30 °C, 300 rpm	2.5 µM, 24 h	AFB1 detoxification ranged from 45.7% to 56.7%	-	-	(Bian et al., 2025) [72]
Dye-decolorizing peroxidase (BsDyP)	*Bacillus subtilis* SCK6; recombinant expression in *Escherichia coli*	Malonate buffer (50 mM, pH 4.0), 30 °C	1.25 U/mL, 48 h	AFB1 detoxification ranged from 50.0% to 76.93%	AFB1-diol	LC–MS/MS	(Qin et al., 2021) [73]
Laccase	*Trametes versicolor* (commercial enzyme)	Acetate buffer (100 mM, pH 6.5), 28 °C, shaking; no mediator	25 U/mL; up to 96 h	AFB1 reduction of approximately 12% after 96 h	Ring-opened AFB1 products, epoxide and dihydroxylated derivatives	LC-MS	(Zaccaria et al., 2023) [74]
Lipase /Protease (commercial)	Humicola lanuginosa	Fungal culture filtrate containing aflatoxins, 30 °C	25–200 U/mL, 12 h	AFB1 detoxification ranged from 35.8% to 81.3%	-	ELISA	(Al-Rajhi et al., 2024) [75]
Lac-W (laccase, multicopper oxidase)	*Weizmannia coagulans* 36D1 (recombinant, expressed in *E. coli*)	pH 9.0, 30 °C, static incubation, no redox mediator	3 U/µg, 24 h	AFB1 detoxification reached 88% in standard system and 92% in feed matrix	AFQ1	HPLC-FLD; UHPLC-MS/MS	(Hao et al., 2023) [76]
Laccase (LAC3, recombinant)	*Saccharomyces cerevisiae*	pH 5.7, 30 °C	3 U/mL, 12–60 h	AFB1 detoxification reached 90.33%	-	HPLC-MS	(Liu et al., 2020) [77]
Laccase (immobilized on BF-NH2)	*Bacillus amyloliquefaciens*	pH 5, 30 °C	0.2 U, 5 h	AFB1 detoxification reached 90% in corn oil	AFQ1	HPLC	(Rasheed et al., 2024) [78]
Dye-decolorizing peroxidase (BaDyP)	*Bjerkandera adusta*	pH 4.0, 30 °C, 1 mM Mn^2+^ or 1-HBT	1 U, 48 h	AFB1 detoxification reached 86.68%	AFB1-diol, AFQ1, 15-OH-ZEN, HZEN, C_15_H_18_O_8_	UPLC-MS/MS	(Shao et al., 2024) [79]
Laccase (ApeLip), Dye-decolorizing peroxidase (KpDyp), Lignin peroxidase (TrcLip), Versatile peroxidase (VPL2)	*Agrocybe pediades, Klebsiella pneumoniae, Trametopsis cervina, Pleurotus eryngii*	pH 9.0, 37 °C	24 h	AFB1 detoxification ranged from 90.06% to 92.91%	AFB1-8,9-dihydrodiol	UPLC-QTOF-MS	(Wang et al., 2025) [80]
DyP (Dye-decolorizing peroxidase)	*Paracoccus* sp. XF-30	30 °C	48 h	AFB1 detoxification reached 71.63%	AFQ1	HPLC	(Hu et al., 2024) [81]

**Table 4:** Experimental models for aflatoxin B1 detoxification: biological agents or enzymes applied, tested matrices, and outcomes. Bacterial names were standardized using currently accepted nomenclature.

Experimental Application	Biological Agent/Enzyme	Tested Matrix	Outcomes	Reference
Bread making (fermentation + baking)	*Lactiplantibacillus plantarum* B3 lyophilized	AFB1-contaminated maize flour	AFB1 detoxification reached 55.0% compared with contaminated control flour	(Escrivá et al., 2023) [33]
Biocontrol during storage	*Bacillus subtilis* E11	Dried red chili (*Capsicum annuum* L.) artificially inoculated with *A. flavus*	Almost complete inhibition of fungal growth; significant reduction in AFB1 after 10 days compared with control	(Yuan et al., 2023) [22]
Biocontrol during grain storage	*Streptomyces exfoliatus*	Wheat grains	Significant suppression of *Aspergillus flavus* growth and sporulation; AFB1 reduced to undetectable or very low levels compared with untreated controls	(El-Shanshoury et al., 2022) [43]
Fermentation-based detoxification	*Enterococcus faecium* HB2-2	AFB1-contaminated peanut meal	AFB1 detoxification ranged from 47.7% to 82.9%, depending on the solid-to-liquid ratio	(Feng et al., 2024) [52]
Solid-state fermentation	*Lactobacillus helveticus* FAM22155	Wheat bran	AFB1 detoxification reached 86–89% after 48 h; activity mainly attributed to proteinaceous metabolites produced during fermentation	(Zhang et al., 2021) [56]
Sourdough fermentation	*L. plantarum* ATCC 8014 and *L. rhamnosus* ATCC 7469	AFB1-spiked sourdough (10 µg/kg)	Maximum AFB1 adsorption occurred with thermosonication-treated co-culture during proofing (37 °C, 24 h)	(Abedi et al., 2022) [32]
Traditional fermentation (1 year)	*Bacillus albus* YUN5	Doenjang	Total aflatoxins decreased to 6.04 ± 3.17 µg/kg (*B. albus*) and 16.91 ± 0.00 µg/kg (cell-free supernatant) after 12 months	(Kumar et al., 2023) [48]
Washing wastewater treatment	Immobilized CotA laccase	Wastewater generated from washing food matrices (rice, red ginseng, etc.) and traditional Chinese medicinal materials	Complete removal of AFB1 after 6 h	(Zhang et al., 2025) [70]
Post-harvest treatment	Lactic acid bacteria probiotics (yogurt-derived)	Naturally contaminated corn, rice and wheat	AFB1 detoxification reached 52.28% (corn), 83.03% (rice), and 77.22% (wheat) after 7 days	(Zahra et al., 2025) [82]
Chocolate fortification	Activated charcoal + *Lacticaseibacillus rhamnosus* + *Saccharomyces cerevisiae* (tri-mix)	Dark chocolate	AFB1 detoxification ranged from 90.2% to 96.8%, depending on pH and incubation time	(Hamad et al., 2023) [64]
In vitro digestion (gastric, duodenal and colonic phases)	*L. curvatus* 14, *P. pentosaceus* 4, *B. firmus* 6	Extract from naturally contaminated maize flour	AFB1 detoxification reached 72.3% (*P. pentosaceus* 4) and 69.7% (*L. curvatus* 14) during the colonic phase	(Rafai et al., 2025) [55]
Mycoremediation during mushroom cultivation	*Pleurotus ostreatus*	Commercial mushroom substrate supplemented with maize (1:1)	AFB1 detoxification ranged from 53% to 87% in spent substrate, negligible AFB1 detected in fruiting bodies	(Zapaśnik et al., 2025) [58]
In vitro gastrointestinal digestion	Indigenous probiotic bacteria and yeasts	AFB1 solution under gastric and intestinal simulated fluid	AFB1 detoxification reached up to 31.14% during digestion	(Zolfaghari et al., 2020) [34]
Silage fermentation	*Bacillus amyloliquefaciens* YUAD7	Alfalfa silage artificially contaminated with AFB1	AFB1 concentration decreased from 100 µg/kg to 1.7 µg/kg after 48 days	(Tang et al., 2024) [19]
Feed detoxification (GI simulation)	*Sporidiobolus pararoseus* KM281507	Poultry feed (GI simulation)	AFB1 detoxification reached 93%	(Tapingkae et al., 2022) [66]
Food matrix treatment	*Microbacterium proteolyticum* B204 (cell-free supernatant)	Peanuts, corn, cheese	AFB1 detoxification reached78.0% (peanuts), 83.3% (corn), 58.7% (cheese) after 24 h	(Yan et al., 2022) [57]
Food fermentation model	*Geotrichum candidum* XG1	Red pepper (*Capsicum annuum*)	AFB1 detoxification reached 83.0% after treatment	(Yang et al., 2024) [62]
Feed biopreservation	*Levilactobacillus brevis* DN-1	Artificially contaminated peanut cake and sunflower cake	Complete inhibition of AFB1 production after 5 days, 96.4% detoxification after 10 days in peanut cake, complete inhibition after 10 days and 92.7% detoxification after 30 days in sunflower cake	(Wang et al., 2024) [31]
Enzymatic treatment of contaminated feed	Lac-W laccase	Naturally contaminated corn cob	AFB1 concentration decreased from 63 ppb to 5 ppb (92% detoxification)	(Hao et al., 2023) [76]
Food model (co-incubation)	Water kefir grains	Cow milk, Longjing tea infusion, Tieguanyin tea infusion, black tea infusion	AFB1 detoxification ranged from 54.90% to 58.85%	(Ouyang et al., 2024) [83]
Solid-state fermentation	*Rhodococcus turbidus* PD630	Contaminated corn, wheat, and peanut flour	AFB1 detoxification reached 64.17–67.66% in wheat and corn, and 56.74% in peanuts	(Liu et al., 2023) [42]

## Data Availability

No new data were created or analyzed in this study.

## Supplementary Materials

The following supporting information can be downloaded at: https://www.mdpi.com/article/10.3390/toxins18070313/s1, File S1: Completed PRISMA 2020 Checklist; File S2: Completed PRISMA 2020 for Abstracts Checklist.

## Abbreviations

The following abbreviations are used in this manuscript:

AFB1	Aflatoxin B1
AFB2a	Aflatoxin B2a
AFQ1	aflatoxin Q1
DyP	Dye-decolorizing peroxidase
ELISA	Enzyme-linked immunosorbent assay
EPS	Exopolysaccharide
HPLC	High-performance liquid chromatography
HPLC-FLD	High-performance liquid chromatography with fluorescence detection
HPLC-HRMS	High-performance liquid chromatography high-resolution mass spectrometry
HPLC-MS	High-performance liquid chromatography–mass spectrometry
HPLC-QTOF-MS	High-performance liquid chromatography quadrupole time-of-flight mass spectrometry
HPTLC	High-performance thin-layer chromatography
LC-HRMS	Liquid chromatography high-resolution mass spectrometry
LC-MS	Liquid chromatography–mass spectrometry
LC-QTOF-MS	Liquid chromatography quadrupole time-of-flight mass spectrometry
PRISMA	Preferred Reporting Items for Systematic Reviews and Meta-Analyses
UHPLC	Ultra-high-performance liquid chromatography
UHPLC-MS/MS	Ultra-high-performance liquid chromatography tandem mass spectrometry
UPLC	Ultra-performance liquid chromatography
UPLC-QTOF-MS	Ultra-performance liquid chromatography quadrupole time-of-flight mass spectrometry
